# Nanosized κ-Carbide and B2 Boosting Strength Without Sacrificing Ductility in a Low-Density Fe-32Mn-11Al Steel

**DOI:** 10.3390/nano15010048

**Published:** 2024-12-30

**Authors:** Changwei He, Yongfeng Shen, Wenying Xue, Zhijian Fan, Yiran Zhou

**Affiliations:** 1 Key Laboratory for Anisotropy and Texture of Materials (Ministry of Education), School of Materials Science and Engineering, Northeastern University, Shenyang 110819, China; 2 The State Key Lab of Rolling & Automation, Northeastern University, Shenyang 110819, China; 3 Key Laboratory of Neutron Physics and Institute of Nuclear Physics and Chemistry, China Academy of Engineering Physics, Mianyang 621999, China; 4 Analytical and Testing Center, Northeastern University, Shenyang 110819, China

**Keywords:** lightweight steel, κ′-carbides, B2, strengthening, ductility

## Abstract

High-performance lightweight materials are urgently needed because of energy savings and emission reduction. Here, we design a new steel with a low density of 6.41 g/cm^3^, which is a 20% weight reduction compared to the conventional steel. The mechanical properties and microstructures of the steels prepared with different routes are systematically explored by utilizing uniaxial tensile testing and transmission electron microscopy. The steel processed by cold rolling and recrystallization annealing at 950 °C for 15 min shows an ultra-high yield strength of 1241 ± 10 MPa, while retaining a good ductility of 38 ± 1%. The high yield strength is mainly related to the synergistic precipitation strengthening introduced by nanoscale B2 and κ′-carbides. It is encouraging to notice that the yield strength increased without scarifying ductility, compared to the ST steel. The key reason is that the high strain hardening rate is activated by combined factors, including the blockage of numerous twins and nanoscale B2 to the dislocation movements, and dynamic slip band refinement. This study is instructive for concurrently enhancing the strength and ductility of austenitic lightweight steels with fully recrystallized grains and dual nano-precipitates.

## 1. Introduction

With the urgent requirement to save energy and protect the environment, one of the effective solutions is to reduce the weight of alloys while maintaining high yield strength to ensure their safety in structural applications, such as aerospace and transport [1,2,3]. Among them, Fe-Mn-Al-C lightweight steel has attracted considerable attention because of its low density and excellent mechanical performance [4,5]. Chen [6] and Zambrano [7] have presented a broad overview of the current technical level of such alloys. The density of this kind of alloy can be significantly reduced by the additions of Al and C lightweight elements, where the density is decreased by about 1.3% via 1% Al addition [8,9]. High Al and C contents in Fe-Mn-Al-C lightweight steel also facilitate the precipitation of nanoscale κ′-carbides with the L1_2_ structure [10]. In general, κ′-carbides are generated by spinodal decomposition during solution treatment and aging and quenching, which usually precipitate in the austenitic grains. In particular, the κ′-carbides have a nanosized cubic morphology and maintain a coherent relationship with the austenitic matrix [11], hence substantially enhancing the strength of Fe-Mn-Al-C steel. However, cooling at the slow rates after solid solution and prolonged aging at high temperatures (over 650 °C) could result in the precipitation of κ*-carbides at the grain boundary, thereby reducing the plasticity as well as the yield strength of the material [5]. In 2015, Kim et al. [12] obtained a combination of an ultra-high yield strength and work-hardening rate by adding the Ni element, which promotes the precipitation of Ni-Al type intermetallic compounds (B2) in Fe-15Mn-10Al-0.8C (wt.%) duplex steels, and modulates the distribution of B2. The results indicate that the brittle intermetallic compound B2 can be used as a reinforcing phase to improve the strength and work hardening rate. Subsequently, a series of studies have been carried out on the deformation mechanisms, B2 precipitation phase morphology modulation [13,14], and impact and spalling behavior of Fe-Mn-Al-C low-density steels containing Ni [15,16]. However, it is still challenging to concurrently modulate the precipitation behavior of κ′-carbide with the B2 phase.

Increasing the yield strength of Fe-Mn-Al-C steels while maintaining excellent plasticity remains a great challenge. In Fe-Mn-Al-C steels, in addition to regulating the internal precipitation of the alloy, different rolling processes were frequently introduced in combination with recrystallization annealing to optimize the combination of strength and ductility [17]. Very recently, heterogeneous structures (HSs) have become a hot topic as they proved to be an effective way to increase strength without significantly reducing ductility [18,19,20]. During the construction of an HS in Fe-Mn-Al-C steels, numerous studies have been carried out to design the layered HS by introducing cold rolling and partial recrystallization annealing, and to modulate κ-carbide and B2 precipitation for obtaining an excellent combination of yield strength and ductility [21,22]. However, recent studies have indicated that the partially recrystallized structure may not be ideal for designing an HS [23]. This is because it retains a high density of dislocations in the non-recrystallized regions, thus limiting the movements and storage of the dislocations when the specimen is loaded. As a result, the work-hardening capacity is reduced [24]. Encouragingly, it has been reported that excellent mechanical properties can be achieved by modulating κ-carbide and B2 precipitation behaviors in austenitic steels with a fully recrystallized microstructure [4,9,25], thus leading to a high yield strength and good ductility [13]. Nevertheless, the results showed that the obtained B2 took over the finer κ′-carbide to occupy the main region of the austenitic grains, which often led to a decrease in yield strength. Moreover, it was difficult to avoid B2 aggregating and coarsening at grain boundaries, which would be detrimental to plasticity. Therefore, it is still challenging to obtain high yield strengths in austenitic low-density steels with a fully recrystallized microstructure and retain good ductility.

To hurdle the dilemma for simultaneous precipitations of κ-carbides and B2 precipitates and the poor deformation capability of uncrystallized structures with fabulous dislocations in austenitic steels, this study proposes a new strategy to generate a fully recrystallized structure with dispersedly distributed κ-carbides and B2 precipitates, aiming to obtain a good combination of an ultra-high yield strength and ductility in the low-density austenitic steels. This should provide a referring target for the development of low-density austenitic steels.

## 2. Experimental Methods

### 2.1. Materials Preparation

**Figure 1:**
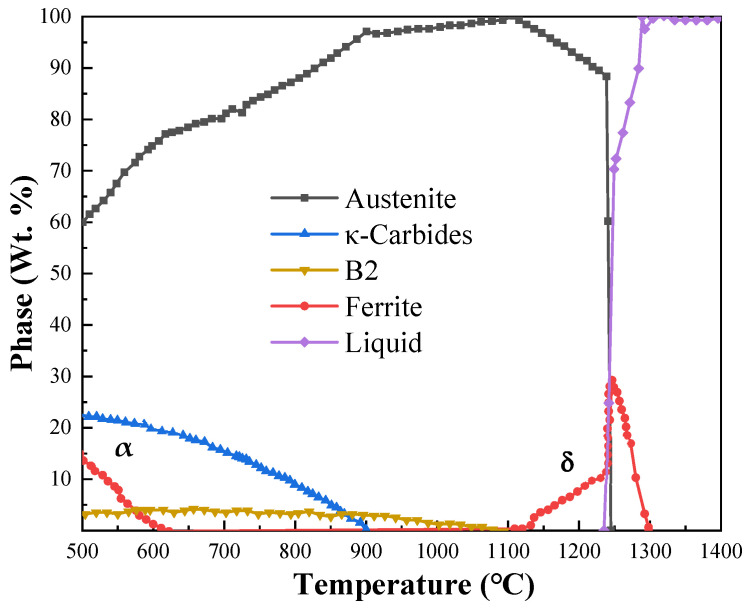
Phase distributions in Fe-32Mn-11Al-1.4C-3Ni steel simulated by JmatPro V10.0.

The chemical compositions of steel used in this study are as follows (in wt.%): 32 Mn, 11 Al, 3 Ni, 1.4 C, 0.1 Ce, and balanced with Fe. A 20 kg ingot was cast via a vacuum induction melting furnace protected with an Argon atmosphere. The JmatPro simulation shows that the density is 6.41 g/cm^3^ for the steel, which is equivalent to 78.6% of the value of conventional austenitic steel (8.15 g/cm^3^). The ingot was hot-forged and air-cooled to room temperature. Based on the equilibrium phase diagrams (Figure 1), the δ phase (high-temperature ferrite) preferentially occurs once the temperature is over 1100 °C, which is detrimental for the mechanical performance. Therefore, a billet with dimensions of 80 × 45 × 35 mm^3^ was cut from the forging and was hot-rolled at 1050 °C for 8 passes with a total reduction of 85% to obtain a plate with a thickness of 5 mm, named HR steel. The finishing temperature of the rolling processing was 950 °C. Subsequently, the HR plate was divided into two parts and one of them was conducted solution treatment (ST) at 1050 °C for 4 h and air-cooled to room temperature. The resultant plate was divided into two parts; one was named ST steel while the other was further cold-rolled for 30 passes with a total reduction of 60%, obtaining a plate with a thickness of 2 mm. Then, the plate was heat-treated at 950 °C for 15 min to promote complete recrystallization, and the resultant was named ST-CA steel.

### 2.2. Microstructure Characterization

The crystallographic structures were characterized by using a Rigaku Smart-lab X-ray diffractometer (XRD, Nippon Rigaku Corporation, Tokyo, Japan) equipped with Cu Kα radiation, with the 2*θ* ranging from 30 to 100° and a scanning speed of ~0.07 s^−1^, operated at 40 kV and 200 mA. The specimens were polished by using diamond paste (*d* = 2.5 μm) until the surface was smooth, and then electrolytically polished in a solution composed of 10% perchloric acid and 90% ethanol at 20 V for 40 s. Subsequently, the microstructure of the specimens was further observed by using the JSM-7001F field-emission scanning electron microscope (SEM, Nippon Electronics Corporation, Tokyo, Japan) in conjunction with the HKL electron backscatter diffraction (EBSD) detector. Transmission electron microscopy (TEM) observations were performed by using an FEI Tecnai G2 F20 microscope (FEI Corporation, Hillsboro, OR, USA) operating at an accelerating voltage of 200 kV, and disks with a thickness of 45 nm and a diameter of 3 mm were prepared by using a twin-jet polisher operated at 25 V in a 10% perchloric acid alcohol solution.

### 2.3. Mechanical Properties Testing

The dog-bone-shaped samples with dimensions of 15 × 5 × 1.5 mm^3^ were cut along the rolling direction (RD) of the prepared plates by using an electrical discharge machine. Tensile tests were performed on an AG-XPLUS 100 KN (Shimadzu, Kyoto, Japan) hydraulic universal mechanical testing machine at a strain rate of 1 × 10^−3^ at room temperature. The strain levels were calibrated via a video extensometer. Three samples of each steel were used for tensile testing, and the average value was selected for representing the mechanical performance of the identical steel.

## 3. Results

### 3.1. Microstructure of the Three As-Prepared Steels

**Figure 2:**
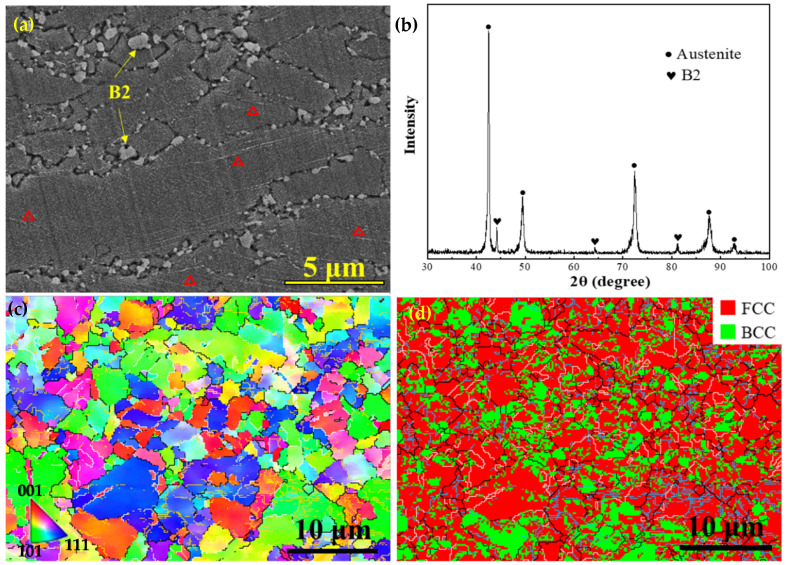
Microstructure of the HR steel: (**a**) SEM image with red triangles indicating intracrystalline nano-B2, (**b**) XRD pattern, (**c**) IPF map, and (**d**) the corresponding phase distribution map with the FCC (austenite) and BCC (B2) structures in red and green, respectively.

Figure 2a is a SEM image of the HR steel, and the austenitic grains exhibit an elongated morphology. A remarkable feature is that extensive micron-sized particles rest at the grain boundaries. Meanwhile, a few fine particles can also be observed inside the grains and the distribution is mainly linear. The XRD pattern revealed that the particles are the B2 phase (Figure 2b). The EBSD inverse pole figure (IPF) shows that the grain morphology is homogenous and there is no obvious texture, where the grains with (111), (101) and (001) orientations are indicated in blue, green, and red, respectively (Figure 2c). The statistical results showed that the average grain size is 5 ± 0.5 μm. The corresponding phase distribution map reveals that the B2 phase has a BCC crystal structure and mainly distributes near the grain boundaries, and the ratio of the B2 phase is as high as 27% (Figure 2d). In addition, the high-angle grain boundaries (HAGBs, >15°) and twin boundary (Ʃ3 60° <111>) are marked by black and white lines, respectively, while low-angle grain boundaries (LAGBs, 2–15°) are indicated by purple lines. The twins with the Ʃ3 boundaries can be regarded as annealing twins. During heat treatment, the intra-crystalline stress caused by the deformation in different regions of the material will result in the formation of annealing twins. Thus, the appropriate annealing temperature and time can induce the formation of twins. It can be seen that the fraction of LAGBs accounts for 40.1%, suggesting that a large number of sub-grain boundaries still exist in the HR steel and the target of a fully recrystallized microstructure has not been reached. In particular, the ratio of twin boundaries accounts for 35.7%, which means that the majority of HAGBs are twin boundaries in the HR steel.

Based on the equilibrium phase diagram (Figure 1), it can be seen that the dissolution temperature of the B2 phase is about 1050 °C. Hence, it is reasonable to observe coarse B2 particles at the austenite grain boundaries in the HR steel, as indicated by the yellow arrows (Figure 2a). Meanwhile, the substantial and fine particles can also be seen in the lines inside of the grains, indicated by the red triangles. In general, the coarse B2 particles are difficult to be eliminated by the annealing treatment at low temperatures and tend to agglomerate and roughen at the grain boundaries, which further impairs the ductility of the alloy [14]. Hence, it is necessary to eliminate the B2 particles at the grain boundaries via solution treatment of the HR steel.

**Figure 3:**
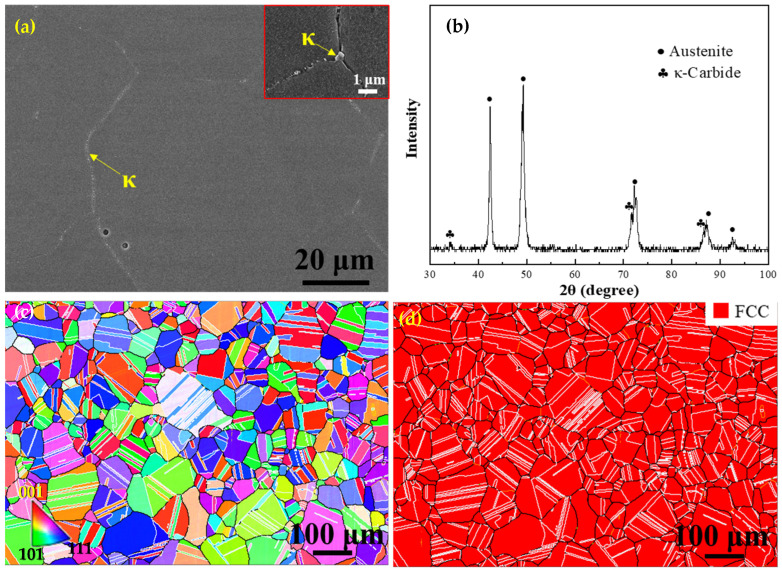
Microstructure of the ST steel: (**a**) SEM image with an inset showing the defects and κ-carbides on grain boundaries, (**b**) XRD pattern, (**c**) IPF map, and (**d**) the corresponding phase distribution map.

Figure 3 illustrates the microstructural characteristics of the ST steel. The SEM image shows the presence of numerous fine precipitates at the grain boundaries, and a high-resolution image (inset at right top) exhibits the defects and κ-carbides on GBs (Figure 3a). The corresponding XRD pattern confirmed the existence of κ-carbides in addition to austenite peaks (Figure 3b). This means that the precipitates at the grain boundaries and inside the grains are actually κ-carbides. The typical microstructural features of the ST steel were further characterized by EBSD (Figure 3c), and one can distinctly see a great number of annealing twins in the grains accompanied with significant increases both in the number of twins and in the size of grains compared to the HR steel. The reason for the increasing number of twins should be attributed to the fact that annealing twins are formed in the recovery stage. Due to large deformation, there were still many deformation areas in the HR steel, which was not conducive to the formation of annealing twins. Thus, it is reasonable to notice that the content of twins in the HR steel is less than that in the ST steel.

Figure 3d demonstrates that there is only the FCC phase (in red) in the ST steel, which is consistent with the equilibrium phase simulation (Figure 1). Evidently, the solution treatment promoted the re-dissolution of the precipitates. The statistic results revealed that the average austenite grain size of ST is about 42 ± 1.5 μm (100 μm for excluding twins, Figure 3c,d). In addition to the formation of extensive twins, the solution treatment also promoted recrystallization because the fraction of LAGBs is distinctly small (~3%) in the ST steel. In contrast, the proportions of HAGBs (black and white lines) and twin boundaries (white lines) were as high as 97% and 66%, respectively (Figure 3d). It is noteworthy that κ-carbide precipitates are particularly fine, making it challenging to visualize due to the low resolution of EBSD.

**Figure 4:**
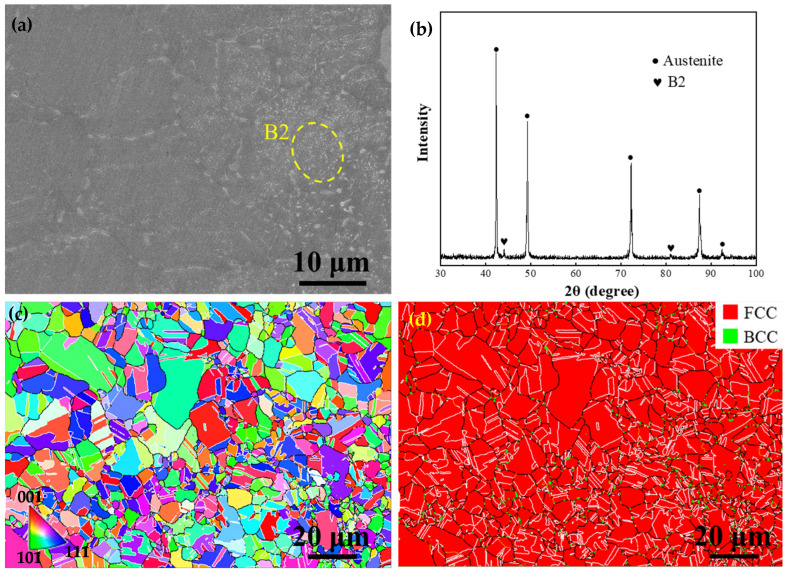
Microstructure of the ST-CA steel. (**a**) SEM, (**b**) XRD results of ST-CA, (**c**) IPF map, (**d**) the corresponding phase distribution map with the FCC (austenite) and BCC (B2) structures in red and green, respectively.

The SEM micromorphology of the ST-CA steel is shown in Figure 4a, and the austenitic grains are relatively small compared to the ST steel. In addition to the extensive and bright particles inside a few austenitic grains, a small amount of granular precipitates occurred at the grain boundaries. The corresponding XRD pattern indicated that the B2 phase exists in the ST-CA steel (Figure 4b). Hence, it can be determined that the precipitates in the ST-CA steel are B2 (marked by a yellow circle, Figure 4a).

Based on the IPF map (Figure 4c) and the phase distribution image (Figure 4d), full recrystallization was achieved in the ST-CA steel; hence, the majority of the grains show the equiaxed shape, accompanied with numerous annealing twins. Nevertheless, the distribution of grain sizes is bimodal, where the average size of small grains is 3 μm, while that of large grains is approximately 7.5 μm, leading to an average grain size of 6 ± 0.2 μm. In comparison with the ST steel, the grain sizes were significantly reduced in the ST-CA steel. The phase distribution map shows that a small amount of precipitates with a BCC structure occurred at the grain boundaries (Figure 4d). It is noticeable that the nanoscale precipitates inside the austenitic grains were unrecognizable in the phase distribution map due to the low resolution of EBSD. In addition, the percentage of LAGBs is only 1.9% and that of HAGBs is as high as 98.1%, in which the fraction of twin boundaries accounts for 58.5% (Figure 4d).

### 3.2. Mechanical Properties

**Figure 5:**
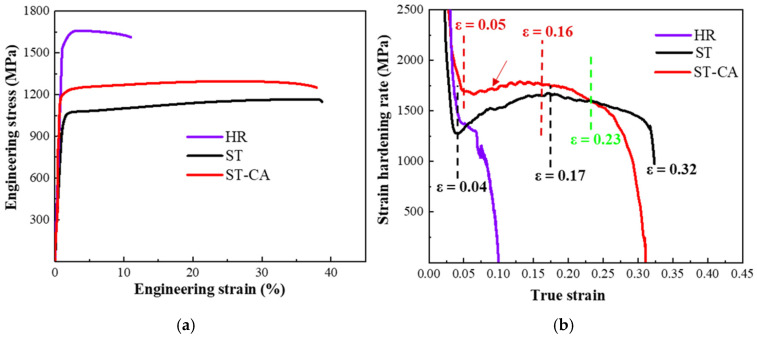
Mechanical properties of the Fe-32Mn-11Al-C steels at different states. (**a**) Engineering stress–strain curves and (**b**) strain-hardening rate curves.

Figure 5a shows the typical engineering stress versus strain curves for the three steels. Despite the high yield stress of 1586 MPa and ultimate tensile stress of 1660 MPa for the HR steel, it has a low ductility of 12 ± 1%. In contrast, the ST steel exhibits a significantly improved ductility of 39 ± 1%; however, the yield stress and ultimate tensile stress decrease to 1074 ± 10 MPa and 1164 ± 15 MPa, respectively. Intriguingly, the yield stress and ultimate tensile stress of the ST-CA steel are distinctly higher than the ST steel, increasing by 167 MPa and 132 MPa, respectively, while retaining a comparable ductility.

**Figure 6:**
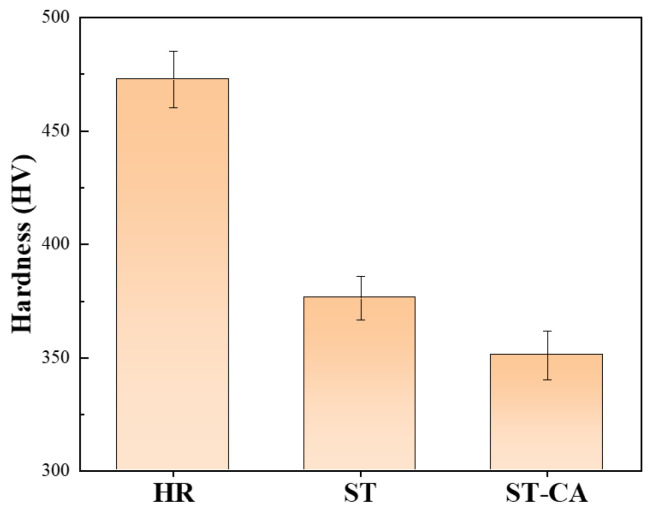
Microhardness values of the three steels.

From the strain-hardening rate curves (Figure 5b), one can clearly see that the strain-hardening rate of the HR steel is particularly low and continuously decreases with increasing strain among the three steels. In contrast, the strain-hardening rate curves of the ST and ST-CA steels reveal a similarly “hump-shaped” shape, i.e., a significantly rising stage after the yield point. This means that the two steels had similar strain-hardening behaviors and that the extra-hardening capability played a key role for the two steels in the stages after the yield. For example, an obvious increase in the strain-hardening rate appears when the strain increases from 0.05 to 0.23 in the ST-CA steel. Meanwhile, a similar scenario can be seen within the strain range from 0.04 to 0.23 for the ST steel. Nevertheless, the difference is that the strain-hardening rate of the ST-CA steel is higher than that of the ST steel in the early stage (ε_t_ < 0.23). Another similar characteristic is that the serrate flow can be seen at the strain-hardening rate curves of the two steels, which should be closely associated with the presence of precipitates in the austenite crystal [25,26,27]. In addition, microhardness is one of the important indicators to characterize the mechanical properties related to the intracrystalline precipitation of alloys. It has been reported that the microhardness values of fully recrystallized steels were closely correlated with the intracrystalline precipitation [5]. Specifically, the higher the size and volume fraction of κ′-carbides, the higher the microhardness value of the austenitic matrix [27]. Here, the microhardness of the ST-CA steel is distinctly lower than that of the ST steel (Figure 6). Hence, the ST-CA steel should contain less κ′-carbides than the ST steel. On the other hand, HR steel has significantly high microhardness when compared with the other two steels. The pivotal reason can be due to the partial recrystallized state and the formation of substantial B2 particles in the HR steel, while the B2 particles are scarce in the ST and ST-CA steels after annealing at high temperatures (Figure 2, Figure 3 and Figure 4).

## 4. Discussion

### 4.1. Precipitation Behavior

**Figure 7:**
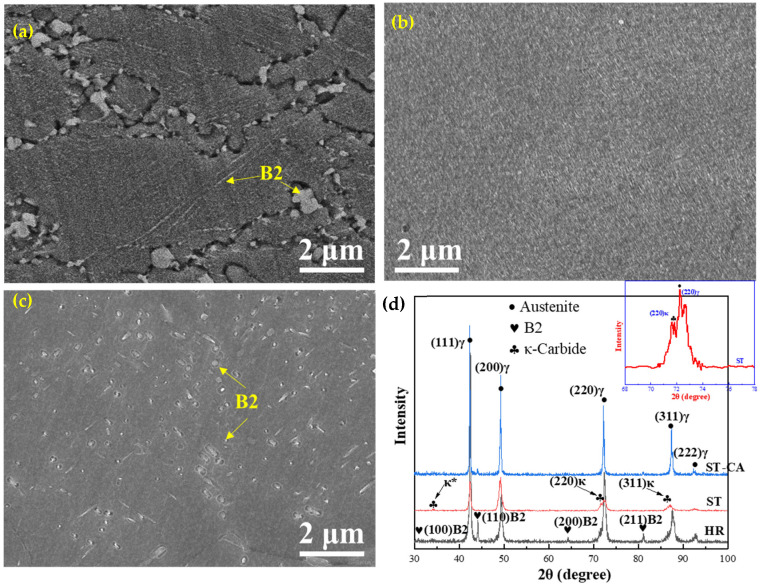
Precipitation states in the different steels: (**a**) HR, (**b**) ST, and (**c**) ST-CA. (**d**) The corresponding XRD patterns. κ* denotes κ-carbides at grain boundaries.

As above mentioned, the HR, ST, and ST-CA steels have significantly different microstructures, especially in the type, morphology, size, and distribution of precipitates (Figure 7a–c). Hence, it is conceivable that the variation of the precipitation behavior should ultimately cause remarkable differences in mechanical performance. The HR steel has a small average grain size (5 μm), and contains large amounts of B2 particles (Figure 2a and Figure 7a). During the hot rolling at 1050 °C, it was conducive to the formation of B2 (Figure 1). Additionally, a considerable number of deformation bands and high-density dislocations were introduced into the austenitic matrix during the rolling deformation, which increased the nucleation sites and nucleation driving force of B2. Consequently, substantially nanoscale B2 precipitates were formed. Meanwhile, the Ni and Al elements enriched at GBs during the growth of austenitic grains should facilitate the precipitation and coarsening of B2 at GBs; hence, the large polygonal B2 particles can be seen at GBs. Indeed, Kim [12] and Geng et al. [15] reported that the precipitation of nanoscale discoidal B2 is favored on the slip bands of the non-recrystallized austenite, whereas polygonal-shaped micron-sized B2 can be produced at the GBs of the fully recrystallized austenite.

The average grain size of the ST steel significantly increases to 42 μm compared to the HR steel. In particular, the percentage of the twinned region reaches a maximum among the three steels (Figure 3c,d). However, the significant difference in the microstructural characteristics of the two steels is the precipitation behaviors within the austenite grain. B2 is replaced by the large amounts of fine and dispersedly distributed κ-carbides in the ST steel, indicated by SEM and the corresponding XRD pattern (Figure 7b,d). This is because the content of Al in the matrix was reduced after the numerous B2 precipitated in the HR steel, thereby reducing the chemical driving force of κ-carbides [8]. Moreover, there still were more deformed structures after the hot rolling, and the large amounts of incoherent B2 particles damaged the coherent state of the austenitic matrix and reduced the nucleation driving force, which was not conducive to the precipitation of coherent κ-carbides. Previous studies showed that κ′-carbides were formed by spinodal decomposition, and the reaction sequence for the precipitation of κ′-carbides in austenitic grains was as follows [28,29]: γ→γ’ + γ″→γ′ + L1_2_→γ′ + κ′. Herein, (1) the spinodal decomposition evokes the partitioning of C and Al in the austenite, resulting in the decomposition of the high-temperature austenite (γ) into two low-temperature austenite, i.e., the solute-poor (C and Al) phase γ’ and the solute-rich phase γ″. (2) Upon further cooling, γ″ transforms to the L1_2_ phase (short-range ordering, SRO). (3) L1_2_ transforms to κ′-carbide. The intracrystalline κ′-carbides are coherent with the matrix, generally presenting a nanosized cubic morphology. The dispersedly fine κ′-carbide is expected to greatly enhance the strength and hardness of the material, and it is the most important reinforcing phase in the low-density Fe-Mn-Al-C steels [30,31]. The detected κ′-carbides can be regarded as coherent because the (220)_κ_ and (311)_κ_ peaks are almost identical to that of (220)_γ_ and (311)_γ_. However, the widths of the (220)_γ_ and (311)_γ_ half-peaks increase because of the introduction of κ′-carbides, even revealing the (220)_κ_ peak, as indicated by the magnified section (inset in Figure 7d). This is because the precipitation of κ′-carbides increases the lattice constant of the austenitic matrix, leading to an increase in the half-peak width of the austenite peaks (Figure 7d). The greater the volume fraction of κ′-carbides, the greater the difference between the lattice constants of κ′-carbides and austenite matrix, which ultimately causes the fractal distributions of the peaks for κ′-carbides and austenite [29,32]. In addition, the XRD pattern of the ST steel reveals a κ-carbide peak at 2*θ* = 34.3° (Figure 7d), suggesting that the κ-carbide was incoherent with the austenitic matrix, hence being considered to be κ*-carbides at grain boundaries (see Figure 3a). In fact, this has been proved in an Fe-30Mn-8.5Al-2C alloy [31].

It is noteworthy that the reported results related to the effect of κ*-carbides on ductility were extremely controversial [5,33]. In general, the ductility of the alloy did not significantly drop when κ*-carbides were refined to submicron sizes and located at GBs [33]. Although most of the κ*-carbides at GBs in the ST steel are still at the submicron level, the presence of κ*-carbides potentially retards the slip of GBs. Additionally, it should be noted that the half-peak width of austenite in the HR steel is relatively large but it is mainly related to the large internal stress caused by the rolling deformation. No κ′-carbide peak can be discerned for the HR steel (Figure 7d), which was caused by the precipitation of Ni-Al type B2 inside the grains during rolling above 900 °C. The hot rolling reduced the Al content in the austenitic matrix and lowered the chemical driving force for κ′-carbide precipitation. Also, the introduction of numerous dislocations in the HR steel increased the distortion energy of the austenitic matrix, which was not conducive to the precipitation of coherent κ′-carbides. The similar proof can be seen from the study on the austenitic Fe-Mn-Al-C alloys reported by Huang [34].

As above mentioned, the ST steel still has disadvantages such as a large grain size and large size of precipitates at GBs (Figure 3); hence, it was necessary to introduce the recrystallization annealing with a high temperature and short duration during the cold rolling. Thus, the grain sizes were significantly refined, simultaneously, and a few nanosized B2 particles could be promoted to form in the interior of the grains. Such recrystallization nucleation and growth are helpful in inhibiting the aggregation and coarsening of B2 at grain boundaries (GBs). As expected, the average grain size of the ST-CA steel markedly decreases and contains a large number of twins (Figure 4c,d). However, the noticeable difference from the ST steel is that partial and nanoscale B2 particles were deposited inside the grains induced by the low annealing temperature (Figure 7c). Moreover, it is conceivable that the melting defects at the grain boundaries (Figure 2a and Figure 3a) were potentially removed by the extra rolling process. Zargaran [13] and Chen et al. [4] reported that a complete intracrystalline nano-B2 precipitation has been achieved after annealing at 900 °C for 15 min. However, this method is often accompanied by a reduction in yield strength and it was difficult to avoid the aggregation and coarsening of B2 at GBs. In addition, the XRD patterns (Figure 7d) show that the κ′-carbide peaks of the ST-CA steel decrease in comparison with that of the ST steel, suggesting a decrease in κ′-carbide volume fraction in the ST-CA steel. Banis [5] and Chen et al. [29] pointed out that there was a positive correlation between the austenitic matrix microhardness and the volume fraction of κ′-carbides in the Fe-Mn-Al-C steels. The information from XRD (Figure 7d) well matches with the microhardness values in Figure 6, where the ST-CA steel has a low microhardness value compared with the ST steel due to less κ′-carbides. As far as the HR steel is concerned, the high microhardness must be related to the existence of extensive B2 particles (Figure 2d) and the partial recrystallized state without annealing. The reason should be associated with the fact that more NiAl-type B2 particles were precipitated at high temperatures (950 °C), which consumed Al in the austenitic matrix [13,21]. As a result, the chemical driving force of κ′ -carbide nucleation decreased.

### 4.2. Deformation Mechanisms

The ST-CA steel has a combined advantage in terms of the production of strength and total elongation (Figure 5a). As far as the yield strength (σ_y_) is concerned, it can be estimated by the sum of various strengthening effects, as follows [3]:

(1) $$\sigma_{y}=\sigma_{0}+\sigma_{Ss}+\sigma_{dis}+\sigma_{GB}+\sigma_{p}$$

Here, σ_0_ is the frictional stress of austenite (97 MPa) [35], σ_Ss_ is the solution strengthening, σ_Ss_ = 61.3C − 1.5Mn + 20.5Al + 2.9Ni, in wt.% [15], σ_dis_ is the dislocation strengthening, $\sigma_{dis}=M\alpha Gb\sqrt{\rho_{dis}}$, *M* is the Taylor factor of the FCC alloy (3.06), α is a constant (0.2), *G* is the shear modulus (71 GPa), *b* is the Burgers vector (0.26 nm), and *ρ*_dis_ is the dislocation density [1]. Meanwhile, σ_GB_ is the GBs strengthening, i.e., $\sigma_{GB}$ = kd^−1/2^, where k is the Hall Petch coefficient (461 MPa μm^1/2^), and *d* is the average size of austenitic grains [9]. In addition, σ_p_ is the precipitation strengthening, which can be estimated by σ_p_ = σ_y_ − σ_0_ − σ_Ss_ − σ_dis_ − σ_GB_ [36]. Among them, dislocation strengthening in the HR steel is calculated while that in the ST and ST-CA steels are neglected because the complete recrystallization is assumed to finish in the two steels.

**Figure 8:**
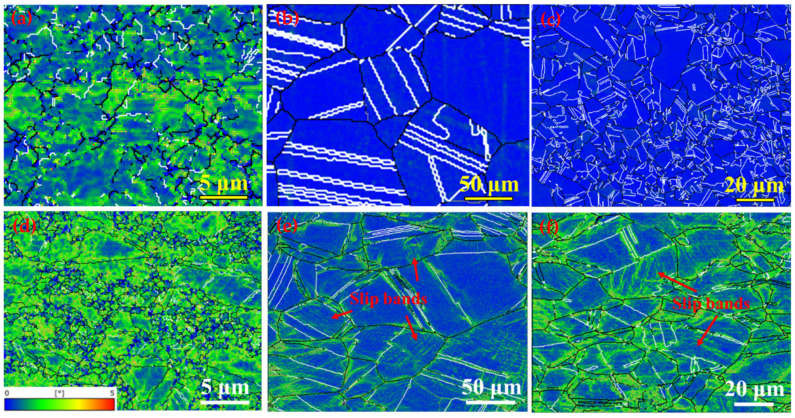
Strain distributions and GBs together with TBs maps in the HR, ST, ST-CA steels, respectively. (**a**–**c**) the as-prepared state, and (**d**–**f**) after tensile deformation. Black lines: GBs, white lines: TBs.

**Figure 9:**
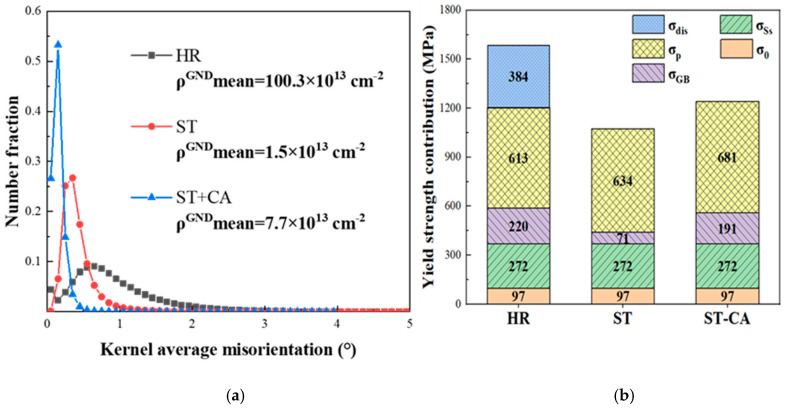
(**a**) KAM distribution before tensile deformation for different process states, (**b**) the contributions of different factors to yield strength for the HR, ST, and ST-CA steels.

As shown in the kernel average misorientation (KAM) distributions before (Figure 8a–c) and after tensile deformation (Figure 8d,e), a large strain already exists in the as-prepared HR steel (Figure 8a); in contrast, few strains can be detected in the ST and ST-CA steels due to the annealing at high temperatures (Figure 8b,c). One distinct feature is that the highest strain level can be seen in the deformed HR steel (Figure 8d), in spite of its poor ductility (Figure 5). Another characteristic is that abundant slip bands occurred in the ST and ST-CA steels, accompanied with the high strain level around them (Figure 8e,f). The formation of multiple slip bands is helpful for enhancing the strain-hardening capability, which is related to dynamic strain refinement [32]. Based on the KAM maps (Figure 8a–c), a geometrically necessary dislocation density (*ρ*^GND^) is derived for the three steels (Figure 9a), respectively. The *ρ*^GND^ values of the ST and ST-CA steels are remarkably smaller than that of the HR steel. Hence, only the contribution of σ_dis_ to the yield strength of the HR steel needs to be considered. Figure 9b shows the contributions of various strengthening factors to yield strengths for the three steels. Evidently, among them, σ_p_ is the most important factor for strengthening the three steels. Additionally, the σ_GB_ value is the highest in the HR steel compared with the ST and ST-CA steels, which is caused by the smallest average grain size of 5 ± 0.5 μm.

It should be pointed out that the σ_p_ in the three steels is not generated by the same reasons. Previous research on the σ_p_ in Fe-Mn-Al-C system low-density steels has indicated that there are two main pathways for precipitation strengthening [37]: (1) The σ_p_ is achieved by precipitating the nanoscale and uniformly distributed dislocation-sharable κ′-carbides because the presence of κ′-carbides has an important impact on the dislocation slip and pile-up during the deformation [33,36]. (2) The precipitation of unshearable nanoscale intracrystalline B2 is conducive to significant reinforcement by the Orowan dislocation bypass mechanism [15,16]. As shown in Figure 2, Figure 3, Figure 4 and Figure 7, the precipitation strengthening is mainly provided by nanoscale B2 in the HR steel; however, that is dominated by the κ′-carbides in the ST steel. Interestingly, the precipitation strengthening in the ST-CA steel resulted from the joint combination of nanoscale B2 and κ′-carbides, owing to the precipitation of nanoscale B2 within many grains. According to the XRD patterns (Figure 7d), the peaks of κ′-carbides in the ST steel are wider than those of the ST-CA steel, even showing the peak separation. This suggests that the size and volume fraction of the κ′-carbides in the ST-CA steel are less than that in the ST steel, and it is consistent with the microhardness values (Figure 6).

Hence, the precipitation strengthening caused by κ′-carbides is less than that in the ST steel (634 MPa), whereas the remainder of the precipitation strengthening can be attributed to the nanosized intragranular B2 particles in the ST-CA steel. It has been reported that the precipitation of nanoscale B2 within the austenite grains enhanced the dislocation bypassing mechanism due to the unshearable nature, thus not only providing an additional yield strength but also contributing to the extra strain-hardening rate [3,9,30]. Here, the strain hardening rate of the ST-CA steel is higher than that of the ST steel at the early stage of deformation (Figure 5b). It can be deduced that the significant improvement in the mechanical properties is caused by the synergistic reinforcement of the B2 and κ′-carbides, the significant reductions in the average grain size, and the elimination of fusion defects at GBs in the ST-CA steel. Despite the κ′-carbide precipitation in the ST steel, the average grain size is large and the grain boundaries contain a few defects related to the incomplete fusion, hence damaging the ductility. In contrast, there are unshearable nano-B2 and κ′-carbides in the ST-CA steel, and the synergistic strengthening from the heterogeneously distributed dual nano-precipitates contributes to increments in the yield strength and tensile strength by 167 MPa and 132 MPa, respectively, while retaining a comparable ductility compared to the ST steel.

**Figure 10:**
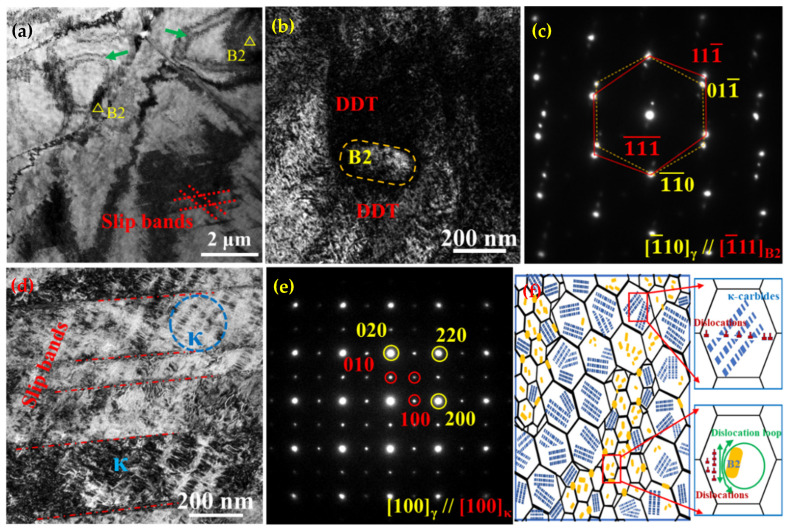
TEM microstructural characterization of ST-CA after tensile deformation, (**a**) TEM morphological characterization, (**b**) microscopic morphology of B2, (**c**) SAED pattern of B2, (**d**) microscopic morphology of κ-carbide, (**e**) SAED pattern of κ-carbide, (**f**) schematic diagrams of precipitation distribution states and precipitation strengthening mechanism of ST-CA.

Figure 10 reveals the TEM morphologies of the deformed ST-CA steel, where the cross-slip feature is indicated by the red lattice (Figure 10a). Meanwhile, the dislocation loops produced by the unshearable nano-B2 can also be observed (green arrow, Figure 10a). From this point of view, the nano-B2 should provide an extra strain-hardening rate during deformation because of the precipitation strengthening. Figure 10b exhibits a typical morphology of B2 in the ST-CA steel, with a size of about 100 nm in width and 200 nm in length. Numerous dense dislocation tangles (DDTs) can be seen around the B2 particle, suggesting the unshearable nature of B2, and the corresponding diffraction pattern is shown in Figure 10c, indicating a K-S orientation relationship between the intracrystalline B2 particle and the austenitic matrix. Figure 10d shows the morphology and distribution of κ′-carbides, which are rock candy-like, extremely fine (20–30 nm), and arranged regularly in the parallel arrays. Also, the plenty of slip bands appear in the regions near the κ′-carbides arrays. Interestingly, one can see the slip bands obviously cut through the κ′-carbides, indicating that the κ′-carbide is dislocation shearable. Figure 10e shows the diffraction pattern of κ′-carbides, suggesting that the κ′-carbides are coherent with the austenitic matrix. Consequently, a schematic illustration is drawn to show the related precipitation strengthening mechanisms in the ST-CA steel (Figure 10f). A fully recrystallized microstructure with a non-uniform grain size is achieved in the ST-CA steel, and the precipitated phases are dominated by substantial and nanosized κ′-carbides together with a few B2 particles. In particular, the B2 particles mainly occur in the region of small grains and its distribution is inhomogeneous. In addition, a few little B2 particles locate at GBs, which hindered the growth of austenite grains during the recrystallization annealing. On the other hand, the numerous precipitations of B2 destroy the co-lattice order of the surrounding austenite, promoting the formation of κ′-carbides around the B2 particles. Due to the unshearable nature, the strengthening mechanism of intracrystalline nano-B2 is dominated by the Orowan mechanism related to the dislocation bypass (Figure 10f). The rest of the austenitic crystal is mainly occupied by κ′-carbides. The κ′-carbides can be cut through by the dislocations during deformation because the size of κ′-carbides is very small and they maintain a coherent relationship with the matrix. It should be pointed out that the nanoscale precipitates in the austenite can strongly block the dislocation movements and contribute to the high yield strength, regardless of whether the dislocations are cutting through the κ′-carbide or bypassing the B2 particles.

In addition to the above advantages, such as low density and high yield strength, another strong point of the ST-CA steel is good ductility, which is closely related to the strain-hardening rate and the deformation mechanisms. Stacking fault energy (SFE) is a key parameter influencing the activation and evolution of a deformation substructure [38,39], and thereby the strain-hardening behavior of materials [40]. The thermodynamic calculation method is the most widely used to estimate the SFE of high manganese steel. According to the computational thermodynamic method [41,42], the SFE of the Fe-32Mn-11Al-1.4C-3Ni alloy at 298 K is about 84 mJ/m^2^. Wang et al. [16] also calculated the SFE to be about 79 mJ/m^2^ for the Fe-29.6Mn-8.8Al-5.2Ni-1.2C wt.% alloy. In general, the strain-induced martensitic phase transformation occurs when the SFE ≤ 20 mJ/m^2^ [43], twins are formed when 20 mJ/m^2^ < SFE ≤ 40 mJ/m^2^ [44], and a partial and complete dislocation slip occurs when the SFE > 40 mJ/m^2^ [45]. In addition, the dislocation slip, such as shear-band induced plasticity (SIP), microband-induced plasticity (MBIP), and dynamic slip band refinement (DSBR), plays a role during deformation when the SFE > 60 mJ/m^2^ [46,47]. Evidently, the deformation mechanism of the steels in this study is mainly controlled by the dislocation slip. The typical feature of DSBR [32], i.e., the grid formed by the cross-slip bands, is observed in the deformed ST and ST-CA steels (red arrows, Figure 8e,f). This is further supported by the TEM observation (red lines in Figure 10a). Under the DSBR mechanism, the dislocations slip to GBs and then accumulate. Subsequently, new non-coplanar slip systems are inspired with the propagation of the deformation, leading to the formation of web-like slip bands. The interactions among the slip bands make the dislocations uniformly distributed inside the grains, which strengthens the grain interiors. As a result, the stress concentration caused by dislocation pile-ups at grain boundaries is effectively weakened, which further enhance the homogenously plastic deformation of the material. Additionally, the formation of web-like slip bands significantly reduces dislocation annihilation, resulting in a high strain hardening rate. In fact, a few new studies [4,48,49,50] have highlighted dynamic slip band refinement as an important strengthening mechanism in high manganese lightweight steels. The key reason is that, in order to coordinate the further strain generated by applied stress, new non-coplanar slip systems need to be activated to release stored dislocations. As a result, a grid-like slip band feature is formed and the slip band distance is refined. This is very helpful for increasing the dislocation density and enhancing the strain-hardening ability of the material. Thus, the strength and toughness of the material is improved. The DSBR mechanism often exhibits a ‘hump-shaped’ work-hardening rate curve [9,32], which is well consistent with the results in Figure 5b. The other ignorable factor is that the massive twin boundaries in the ST and ST-CA steels not only remarkably refine the grain size, but also facilitate the dislocation slip, which effectively inhibits the localization of the strain, hence delaying the occurrence of premature fracture. A similar phenomenon was reported in 1 GPa low density austenitic steel [25].

**Figure 11:**
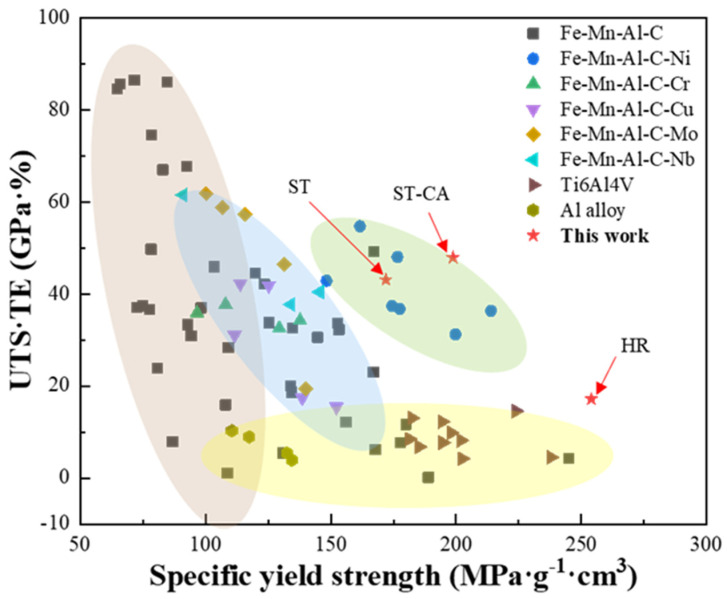
Comparative plot of the mechanical properties of the three steels with the reported Fe-Mn-Al-C systems [9,12,15,17,51,52,53,54,55,56,57,58,59,60,61,62], titanium alloys [63,64,65,66], and aluminum alloys [67,68,69].

Although the ST and ST-CA steels reveal dynamic slip band refinement during the plastic deformation, the ST-CA has a higher strain hardening rate in the early deformation stage compared to the ST steel due to the nano-B2 particles activating a bypassing mechanism (Figure 10a). Furthermore, the small average grain size and low defects in the ST-CA steel significantly impede the crack propagation in the late stage of deformation. Consequently, the dramatic increments of the yield strength and tensile strength are achieved without sacrificing the ductility of the ST-CA steel. Figure 11 summarizes a plot showing the relationship between the mass specific yield strength and tensile toughness of the three steels, compared with the Fe-Mn-Al-C system low-density steels and high-ratio-strength materials, such as titanium alloys and aluminum alloys. Distinctly, the tensile toughness and specific yield strength of the ST-CA steel are significantly improved in comparison with the ST steel, and it is still in an excellent position for a good balance between density and toughness in the current low-density high-strength steels. Despite the significant advantage of a specific yield strength for the HR steel, the production of the ultimate tensile strength (UTS) and total elongation (TE) is poor compared with those of the ST and ST-CA steels.

In summary, the heterogeneously distributed κ′-carbides and unshearable nano-B2 synergistically enhance strength and ductility; however, challenges remain. For example, it is still very challenging to precisely control the precipitations of the κ-carbide and B2 phase and avoid aggregation at GBs. Future studies will focus on optimizing the compositions and regulating the type, size, morphology, and distribution of the precipitates, as well as further improving the microstructural heterogeneity to obtain the optimal mechanical properties.

## 5. Conclusions

In this study, a Fe-32Mn-11Al-1.4C-3Ni-0.1Ce (wt.%) austenitic low-density steel with a high yield strength and good ductility was successfully produced. The microstructure characteristics, mechanical performances, and deformation mechanisms were systemically explored, and the following conclusions can be drawn:

(1) The precipitation behavior has a significant influence on the mechanical properties. The ductility of the HR steel is seriously damaged due to more B2 at grain boundaries. In contrast, there are massive κ′-carbides in the ST steel, hence inducing a yield strength of 1074 MPa. Furthermore, the synergistic strengthening of the unshearable nano-B2 and κ′-carbides results in a high yield strength of 1241 MPa for the ST-CA steel, while retaining a good ductility of 38%.
(2) The κ′-carbides and nano-B2 particles in the ST-CA steel interact with dislocations by different mechanisms. A few B2 particles locate at grain boundaries, thus promoting the formation of small grains during the annealing. As a result, the B2 particles mainly aggregate in the small grain region, and the large and intragranular nano-B2 particles (100–200 nm) are incoherent with the austenitic matrix, hence inducing a bypass mechanism. In contrast, inside the larger grains, the fine size κ′-carbides (~30 nm) are coherent with the matrix, allowing dislocations to cut through the cubic nanoparticles.
(3) The ST-CA steel has distinct advantages in the specific yield strength and the production of UTS and TE. The dynamic slip band refinement mechanism promotes continuous hardening while the unshearable B2 provides additional work hardening, resulting in a significant advantage in terms of tensile toughness in the high specific yield strength range of 200 MPa·g^−1^·cm^3^ compared to the reported low-density steels.

## Data Availability

Data are contained within the article.
